# ESGAR consensus statement on MR imaging in primary sclerosing cholangitis

**DOI:** 10.1007/s00330-025-11583-4

**Published:** 2025-04-26

**Authors:** Davide Ippolito, Cesare Maino, Lionel Arrivé, Ahmed Ba-Ssalamah, Roberto Cannella, Alessandro Furlan, Aristeidis Grigoriadis, Martina Pezzullo, Sarah Pöetter Lang, Sabine Schmidt Kobbe, Federica Vernuccio, Maria Antonietta Bali

**Affiliations:** 1 School of Medicine and Surgery, University of Milan-Bicocca, Monza, Italy; 2 Department of Diagnostic Radiology, Fondazione IRCCS San Gerardo dei Tintori, Monza, Italy; 3 Service de Radiologie, Institut Curie, PSL Research University, Paris, France; 4 Department of Biomedical Imaging and Image-Guided Therapy, Medical University, General Hospital of Vienna (AKH), Vienna, Austria; 5 Section of Radiology, Department of Biomedicine, Neuroscience and Advanced Diagnostics (BiND), University of Palermo, Palermo, Italy; 6 Department of Radiology, University of Pittsburgh Medical Center, Pittsburgh, PA USA; 7 Division of Radiology, Department of Clinical Science, Intervention and Technology (CLINTEC), Karolinska Institutet, Stockholm, Sweden; 8 Department of Radiology, Hôpital Universitaire de Bruxelles HUB, Brussels, Belgium; 9 Department of Diagnostic and Interventional Radiology, Lausanne University Hospital (CHUV) and University of Lausanne (UNIL), Lausanne, Switzerland

**Keywords:** Primary sclerosing cholangitis, Magnetic resonance imaging, MRCP

## Abstract

**Objectives:**

To provide a consensus statement and recommendations on MR imaging in primary sclerosing cholangitis (PSC).

**Methods:**

The European Society of Gastrointestinal and Abdominal Radiology (ESGAR) convened a multinational European panel of experts selected based on a literature review and their leadership in the field. A modified Delphi process was adopted to draft a list of statements. For each statement, the panelists indicated the level of agreement using a 5-point Likert scale, where 1 means “no agreement,” 2 means “poor agreement,” 3 means “slight agreement,” 4 means “fair agreement,” and 5 means “complete agreement.” The median score for each statement was collected. The level of evidence was reported according to the Oxford Centre for Evidence-Based Medicine. Descriptive statistics were used to rate agreement levels and the consensus’ internal reliability.

**Results:**

The 12 voting committee members were from Italy (*n* = 4, 33.4%), Austria (*n* = 2, 16.7%), Sweden (*n* = 1, 8.3%), France (*n* = 1, 8.3%), the United States (*n* = 1, 8.3%), Switzerland (*n* = 1, 8.3%), and Belgium (*n* = 2, 16.7%). The final questionnaire consisted of 55 statements. The agreement reached by the expert panel was complete for 23 statements (41.8%), fair for 16 (29.1%), slight for 15 (27.2%), and poor for 1 (1.9%). Statements that received complete agreement were used to structure a reporting template.

**Conclusions:**

This statement paper recommends how and when to perform MRI in PSC patients. A structured reporting template has been created to improve quality care and communication among radiologists and clinicians.

**Key Points:**

***Question*** *A standard MR protocol and the most common imaging features to be reported are fundamental for the correct evaluation of primary sclerosing cholangitis (PSC) patients.*

***Findings*** *Twelve expert radiologists reported which are the most important imaging features and how and when to perform MR in PSC patients.*

***Clinical relevance*** *The identified statements reported in this paper and the structured reporting template are useful for radiologists and clinicians to help correctly manage PSC patients.*

**Graphical Abstract:**

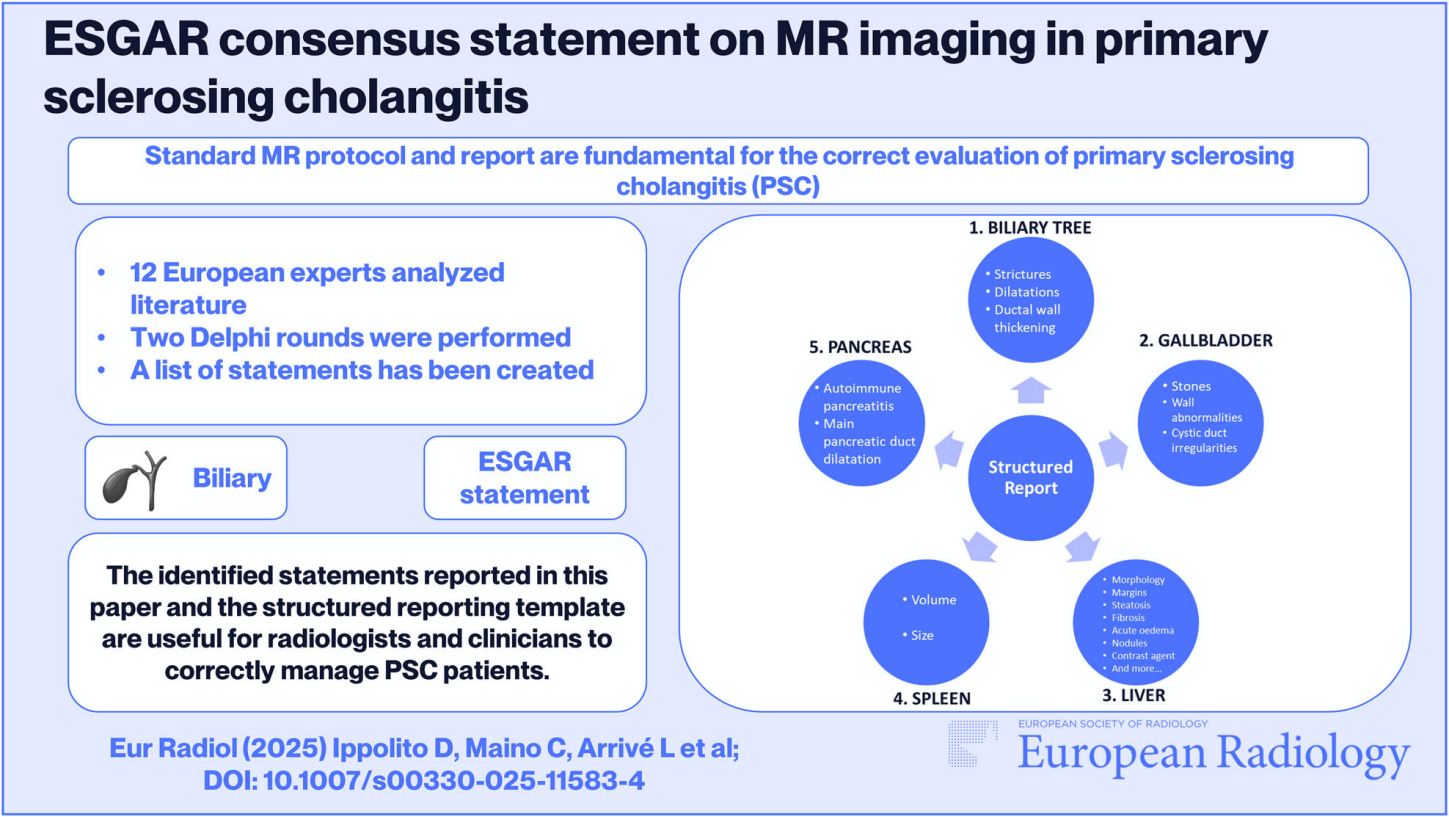

## Introduction

Primary sclerosing cholangitis (PSC) is an idiopathic, heterogeneous, cholestatic liver disease characterized by progressive biliary inflammation and fibrosis that leads to complications including cholestasis and liver failure [1]. “Sclerosing” refers to the stiffening and hardening of the bile duct walls, while “cholangitis” refers to the inflammation of the biliary tree.

This disease typically affects men (60%) with a mean age at diagnosis of 41 years and has an overall incidence of 0 to 1.3 cases per 100,000 individuals per year [2].

Even if the prevalence of PSC varies worldwide, with a peak in the Northern European countries, it seems to be linked to the increased awareness and widespread availability of diagnostic imaging [1].

PSC typically involves both intra- and extrahepatic bile ducts, although, in a small percentage of individuals, only intrahepatic (15–25%) or extrahepatic ducts (5–10%) are affected [3]. There are two established subtypes of PSC: the large-duct subtype affects both small and large bile ducts, and the small-duct subtype affects only small bile ducts, the former subtype having a more favorable diagnosis compared to the latter [4]. The final diagnosis of the small-duct subtype requires liver biopsy since imaging investigations are normal or near-normal. Conversely, liver biopsy is no longer necessary to diagnose large-duct PSC since imaging demonstrates high diagnostic performance. However, if an overlapping condition with autoimmune hepatitis (AIH) or primary biliary cholangitis (PBC) is suspected, liver biopsy is mandatory [5].

Endoscopic Retrograde Cholangiopancreatography (ERCP) has been considered the reference standard for diagnosis in patients with PSC for decades; however, currently, magnetic resonance (MR) imaging with cholangiopancreatography sequences (MRCP) has become the recommended imaging modality for both diagnosis and follow-up of individuals with either suspected or confirmed PSC. Indeed, MRCP is indicated as the first imaging modality for investigating bile duct abnormalities in patients with PSC according to the latest recommendations of the American Association for the Study of Liver Diseases (AASLD) [5] and the European Association of the Study of the Liver (EASL) guidelines [6]. This is primarily because MR with MRCP can provide important information regarding both the biliary tree and liver parenchyma abnormalities non-invasively without ionizing radiation exposure. Moreover, MRCP allows the evaluation of affected bile ducts proximally to a severe stricture that would not have been opacified on ERCP, and this non-invasive diagnostic approach will also prevent endoscopic-related complications [7].

The International PSC Study Group (IPSCSG) has recently suggested reporting standards and guidelines for this disease [8, 9]. Nonetheless, several controversies still exist about the radiological definition of clinically significant strictures and the standardized MR acquisition protocol in patients with PSC. In this setting, there is still an unmet need to standardize image acquisition protocol and structured reporting to promote better communication between healthcare providers and enhance the overall quality of care.

Therefore, the European Society of Gastrointestinal and Abdominal Radiology (ESGAR) Working Group aimed to report findings from this consensus meeting and provide up-to-date practice guidelines for MR imaging acquisition, interpretation and reporting for patients with suspected or diagnosed with large-duct PSC. This position statement is principally based on a review of the international literature, recommendations based on current evidence, and expert opinions. This document includes MR acquisition protocol, technical considerations, patients’ preparation, magnetic field strength, sequences, usefulness of contrast agents, imaging features, including typical findings and complications, and a structured reporting template.

## Methods and consensus process

### Expert panel selection

A call for expressions of interest in participating in the process has been circulated to all active ESGAR members via official email provided by the Society. From those members expressing interest, a panel of twelve members (including the chair) was invited, based on publication records in the field and geographical location, to ensure, as far as possible, appropriate representation across the ESGAR members.

The working group was finally composed of a multinational panel of 12 active and faculty members of ESGAR (D.I., C.M., L.A., A.B.-S., R.C., A.F., A.G., M.P., S.P.L., S.S., F.V., M.A.B.), aiming to represent the Society as widely as possible. As per ESGAR guidelines (esgar.org/guidelines), the ESGAR Research Committee is responsible for starting, directing, and monitoring the development of ESGAR guidelines from the beginning to the end and publishing. ESGAR guidelines are developed by working groups that are dedicated to the guidelines and are composed of ESGAR members who are subject matter experts, according to clinical expertise and previous international publications in the specific field.

### Selection of the Delphi domains and items

All the experts reviewed literature through the main scientific electronic databases, including PubMed, Web of Science, and EMBASE, using search terms such as: “primary sclerosing cholangitis,” “diagnostic imaging,” and “magnetic resonance imaging.”

Case reports were excluded from the first screening procedure. Only English language papers were considered.

To be consistent with the advance of technical improvement, only papers published between January 2000 and June 2021 were included. Considering that the present paper was completed in 2024, references outside the search period are considered consistent only for discussion purposes.

All participants submitted the entire list of analyzed studies, including searching equations for each questioned database. The relevant articles were reviewed and summarized from each panelist. Finally, all expert panel members reviewed the full text of the selected studies, and each of them developed a list of statements and shared them via email.

The study panel was divided into seven subsections: (1) general statements, (2) protocol and patients’ preparation, (3) biliary findings, (4) liver findings, (5) complications, (6) splenic and pancreatic findings, and (7) functional imaging.

Two Delphi rounds were performed. During the first round, each panelist independently contributed to refine the study research draft through online meetings or email exchanges. The panelists’ agreement level for each study research model was tested in the second Delphi through a Google Form questionnaire shared by email.

According to ESGAR recommendations, during the second round, each panelist graded all statements for quality using levels of evidence provided by the Oxford Centre for Evidence-Based Medicine (www.cebm.net). After Delphi rounds, all papers were reviewed from panelists to check and approve levels of evidence.

Finally, according to the final position statement, all panelists approved a structured reporting template for MR draft by the chair of the present paper.

### Statistical analysis

All panelists’ ratings for each statement were analyzed with descriptive statistics.

For each statement, the panelists indicated the level of agreement using a 5-point Likert scale, where 1 means “no agreement,” 2 means “poor agreement,” 3 means “slight agreement,” 4 means “fair agreement,” and 5 means “complete agreement”. The median score for each statement was collected.

All analyses were computed using SPSS (IBM SPSS Statistics v 26.0).

## Results

The final questionnaire was composed of 55 items. All panelists completed the voting for all statements.

The 12 voting panel members were from Italy (*n* = 4, 33.4%), Austria (*n* = 2, 16.7%), Sweden (*n* = 1, 8.3%), France (*n* = 1, 8.3%), US (*n* = 1, 8.3%), Switzerland (*n* = 1, 8.3%), and Belgium (*n* = 2, 16.7%).

Ten out of 12 panel members (83.3%) use 1.5-T MR scanners, and 2 members (16.7%) use 3-T routinely.

Table 1 reports all statements with the respective median score, agreement, and level of evidence according to the Oxford Centre for Evidence-Based Medicine.

**Table 1:** All statements included in the survey

Statement	Median score*	Agreement^#^	Evidence^$^
General statements			
MR should be the first diagnostic modality in patients with suspected or confirmed PSC	5 (*n* = 12, 100%)	Complete	3
MR should be performed before interventions or stent placement	5 (*n* = 11, 91.7%)	Complete	3
In patients with PSC with indeterminate results of MR, a new high-quality MR should be performed in an expert center	5 (*n* = 11, 91.7%)	Complete	5
ERCP should not be performed for the diagnosis of PSC	5 (*n* = 9, 75%)	Complete	3
Protocol and patient preparation			
The minimal recommended magnetic field strength is 1.5 T	5 (*n* = 12, 100%)	Complete	4
Patients should be fasting before the MR examination for at least 4 h	5 (*n* = 11, 91.7%)	Complete	4
Spasmolytics do not need to be administered	5 (*n* = 9, 75%)	Complete	5
T2-hypointense oral contrast agents, such as pineapple or blueberry juice, should be administered before MR	4 (*n* = 7, 58.4%)	Fair	4
The minimum protocol for diagnosis and follow-up must include: T2WI in axial and coronal planes (non-fat suppressed), T1WI IP and OP or DIXON T1, 2D or 3D MRCP	5 (*n* = 12, 100%)	Complete	4
DWI using low and high b-values should be implemented as it provides additional information regarding active inflammation and complications	5 (*n* = 12, 100%)	Complete	4
Administration of intravenous contrast agent should be used to characterize focal liver lesions and parenchymal abnormalities	4 (*n* = 7, 58.4%)	Fair	4
If contrast medium is administered, HBA should be preferred over ECA	4 (*n* = 7, 58.4%)	Fair	4
Biliary findings			
The presence of strictures should be reported as “present” or “absent”	5 (*n* = 12, 100%)	Complete	5
The presence of strictures should be reported as “localized” (= segmental/lobar) or “diffuse”	5 (*n* = 12, 100%)	Complete	5
Terms such as “stenosis” and “dilation” should be preferred to imprecise terms such as “beaded,” “pruned-tree appearance” or “irregularity of bile ducts”	4 (*n* = 7, 58.4%)	Fair	5
Ductal caliber measurement is necessary for stricture(s) grading	4 (*n* = 8, 66.7%)	Fair	5
A dominant stricture is defined as “a stenosis < 1.5 mm in diameter in the CBD”	3 (*n* = 7, 58.4%)	Slight	3
A dominant stricture is defined as “a stenosis < 1 mm in diameter in the right/left hepatic ducts”	3 (*n* = 7, 58.4%)	Slight	3
Wall thickness measurement of CBD should be collected and reported	3 (*n* = 6, 50%)	Slight	5
A high-grade stricture is defined as biliary stricture with ≥ 75% reduction in the caliber	5 (*n* = 10, 83.3%)	Complete	5
A low-grade stricture is defined as biliary stricture with < 75% reduction in the caliber	5 (*n* = 10, 83.3%)	Complete	5
The length of the most severe stricture(s) should be reported	5 (*n* = 10, 83.3%)	Complete	5
Peribiliary enhancement is a consistent finding of PSC	4 (*n* = 9, 75%)	Fair	3
Gallbladder evaluation is mandatory for the detection of mass lesions	4 (*n* = 7, 58.4%)	Fair	2
The definition of functional stricture was recently introduced and needs external validation	3 (*n* = 6, 58.4%)	Slight	3
MIP images acquired during HBP are not recommended for the evaluation of biliary ducts	3 (*n* = 7, 58.4%)	Slight	3
Liver findings			
Liver morphology abnormalities should be concisely reported	5 (*n* = 12, 100%)	Complete	5
Radiological signs of fibrosis should be reported	5 (*n* = 11, 91.7%)	Complete	5
Inflammatory changes should be reported	4 (*n* = 9, 75%)	Fair	5
Reporting and characterizing regenerative nodules, especially macro-regenerative ones, is necessary	4 (*n* = 8, 66.7%)	Fair	5
Peri-portal edema should be reported	3 (*n* = 6, 58.4%)	Slight	5
Presence of venous collaterals should be reported	5 (*n* = 11, 91.7%)	Complete	2
Portal or portocaval lymph nodes are considered typical hallmarks	4 (*n* = 9, 75%)	Fair	5
Portal-vein diameter measurement is necessary	3 (*n* = 6, 58.4%)	Slight	5
Conventional liver MR is considered superior to biopsy in identifying fibrosis and its distribution	3 (*n* = 6, 58.4%)	Slight	4
Complications			
Contrast-enhanced liver MR with MRCP or contrast-enhanced CT should be preferred to invasive ERCP to detect complications	5 (*n* = 12, 100%)	Complete	1
High-grade or severe strictures require endoscopic intervention with brush cytological sampling	4 (*n* = 9, 75%)	Fair	3
MRCP should be considered useful for predicting positive or negative results of endoscopic treatment of stenosis	4 (*n* = 9, 75%)	Fair	3
Annual follow-up with MRCP is the recommended imaging modality for surveillance for malignant complications, development of strictures and decompensation of liver cirrhosis.	5 (*n* = 12, 100%)	Complete	1
Imaging should be combined with serum CA19-9 and alpha-fetoprotein	4 (*n* = 9, 75%)	Fair	3
ERCP should not be used routinely for follow-up	5 (*n* = 12, 100%)	Complete	2
MRCP associated with contrast-enhanced MR is superior to US for the detection of early-stage cholangiocarcinoma in asymptomatic patients	5 (*n* = 12, 100%)	Complete	3
Splenic and pancreatic findings			
IgG4-related sclerosing cholangitis should be ruled out if imaging features of autoimmune pancreatitis are present	5 (*n* = 12, 100%)	Complete	2
Splenic size should be measured during the initial assessment and follow-up	4 (*n* = 8, 66.7%)	Fair	4
Splenic size and volume have a prognostic role in patients with PSC	4 (*n* = 9, 75%)	Fair	2
Splenic length and/or volume increase during follow-up are associated with clinical progression and increased risk of adverse outcome	5 (*n* = 10, 83.3%)	Complete	2
Functional imaging, techniques or scores under investigation			
Functional Liver Imaging Score (FLIS) should be applied in PSC	3 (*n* = 6, 50%)	Slight	3
T1 MRCP stratifies patients according to functional strictures	3 (*n* = 6, 50%)	Slight	3
Relative gadoxetic acid enhancement evaluation may be a prognostic factor in primary sclerosing cholangitis	3 (*n* = 6, 50%)	Slight	3
MRCP severity indexes such as the ANALI score, and transient elastography are complementary for the evaluation of the prognosis of primary sclerosing cholangitis	4 (*n* = 10, 83.3%)	Fair	2
T1 mapping should be implemented as it could provide additional information regarding active inflammation or fibrosis	3 (*n* = 6, 50%)	Slight	3
Magnetic Resonance Elastography (MRE) should be implemented as it provides additional information regarding liver fibrosis	3 (*n* = 6, 50%)	Slight	2
MRE should be used instead of transient elastography due to its higher reproducibility and added value in grading liver fibrosis	3 (*n* = 6, 50%)	Slight	3
Quantitative MRCP metrics, including software such as MRCP+, can be implemented as it provides additional information regarding the severity of PSC	3 (*n* = 6, 50%)	Slight	3
Radiomics analysis can be implemented as it provides additional information regarding active inflammation or fibrosis	2 (*n* = 10, 50%)	Poor	3

* Median score obtained by the final voting session

^#^ Computed considering the number of responses

^$^ According to OCEBM Levels of Evidence—Guyatt et al [42]

The agreement reached by the expert panel was complete for 23 statements (41.8%), fair for 16 (29.1%), slight for 15 (27.2%), and poor for 1 (1.9%).

Table 2 reports the statements which received complete agreement.

**Table 2:** Statements that acquired a complete agreement from the panelists

Protocol and patient preparation	MR should be the first diagnostic imaging modality in patients with suspected PSC	
	MR should be performed before interventions or stent placement	
	In patients with PSC with indeterminate results of MR, a new high-quality MR should be performed in an expert center	
	ERCP should not be performed for the diagnosis of primary sclerosing cholangitis	
	The minimal recommended magnetic field strength is 1.5 T	
	Patients should be fasting before the MR examination for at least 4 h	
	Spasmolytics do not need to be administered	
	The minimum protocol for diagnosis and follow-up must include: T2WI in axial and coronal planes (non-fat suppressed), T1WI IP and OP or DIXON T1, 2D or 3D MRCP	
	DWI using low and high b-values should be implemented as it provides additional information regarding active inflammation and complications	
Biliary	The presence of strictures should be reported as “present” or “absent”	
	The presence of strictures should be reported as “localized” (= segmental/lobar) or “diffuse”	
	A high-grade stricture is defined as biliary stricture with ≥ 75% reduction in the caliber	
	A low-grade stricture is defined as biliary stricture with < 75% reduction in the caliber	
	The length of the most severe stricture(s) should be reported	
Liver	Liver morphology abnormalities should be concisely reported	
	Radiological signs of fibrosis should be reported	
	Presence of venous collaterals should be reported	
Complications	Contrast-enhanced liver MR with MRCP or contrast-enhanced CT should be preferred to invasive ERCP to detect complications	
	Annual follow-up with MRCP is the recommended imaging modality for surveillance for malignant complications, development of strictures and decompensation of liver cirrhosis.	
	ERCP should not be used routinely for follow-up	
	MRCP associated with contrast-enhanced MR is superior to US for the detection of early-stage cholangiocarcinoma in asymptomatic patients	
Spleen and pancreas	IgG4-related sclerosing cholangitis should be ruled out if imaging features of autoimmune pancreatitis are present	
	Splenic length and/or volume increase during follow-up are associated with clinical progression and increased risk of adverse outcome	

### General statements, patient preparation, and MR acquisition protocol

*Statement 1*: MR with MRCP is the recommended imaging modality in patients with suspected or confirmed PSC.

*Statement 2*: MR with MRCP must be performed before any interventions and biliary stent placement.

*Statement 3*: Both 1.5-T and 3-T MR scanners are considered appropriate, depending on local availability and personal preference.

*Statement 4:* A fasting period of at least 4 h before MR examination is recommended. Spasmolytic agents are not recommended. Oral T2-hypointense agents (e.g., pineapple or blueberry juice) can help suppress the signal of the stomach and duodenum, although this has shown fair agreement.

*Statement 5:* Essential MR sequences to be included in the acquisition protocol are coronal and axial T2 weighted imaging (T2WI) with and without fat-suppression, T1 weighted imaging in- and out-of-phase (T1WI IP/OP) or T1WI DIXON, 2D/3D MRCP, and diffusion-weighted imaging (DWI) with at least 3 b-values.

### Biliary findings

*Statement 6:* Biliary strictures must be reported as “present” or “absent,” “single” or “multiple,” and located at the intra- or extrahepatic ducts or both.

*Statement 7:* The severity of the stricture(s) must be assessed by classifying the strictures as “low-“ or “high-grade,” according to the degree of luminal narrowing (< or ≥ 75%).

*Statement 8:* The extent of strictures needs to be described as “localized” in the case of segmental or lobar distribution or “diffuse” when the entire biliary tree is involved.

*Statement 9:* Biliary dilation(s) must be reported as absent or present. In the latter, the affected bile duct caliber needs to be reported.

*Statement 10:* Biliary duct wall thickening, if present, must be reported and measured.

*Statement 11:* The wall thickening behavior on DWI (absence/presence of restriction) and on contrast-enhanced T1WI (absence/presence of enhancement; homogeneous/heterogeneous) must be reported.

*Statement 12:* The presence of intraductal stones must be reported.

*Statement 13:* The description of any abnormalities of the gallbladder, such as focal or diffuse wall thickening and the presence of stones, must be included in the report.

### Findings in liver, spleen, and pancreas

*Statement 14:* Liver parenchyma abnormalities can occur in PSC patients. On unenhanced images (T2WI, T1WI, and DWI), liver morphology (normal/abnormal), liver margins (smooth/irregular), steatosis (absence/presence), fibrosis (absence/presence), acute edema (absence/presence) and regenerative nodules (absence/presence) need to be reported.

*Statement 15:* On contrast-enhanced T1WI, the appearance of focal liver lesions and their suggested diagnosis (benign/malignant), together with associated abnormalities of the portal venous system, need to be described.

*Statement 16:* The spleen’s size and/or volume must be reported.

*Statement 17:* Abnormal changes in the main pancreatic duct must be reported, including the caliber measurements. Pancreatic parenchyma abnormalities suggestive of autoimmune pancreatitis need to be ruled out.

### PSC-related complications

*Statement 18:* MR with MRCP must be considered the first-line imaging modality to investigate patients suspected of benign PSC complications such as infectious cholangitis.

*Statement 19:* Annual follow-up for disease progression and surveillance for malignant complications such as cholangiocarcinoma is highly recommended.

### Reporting template

**Fig. 1:**
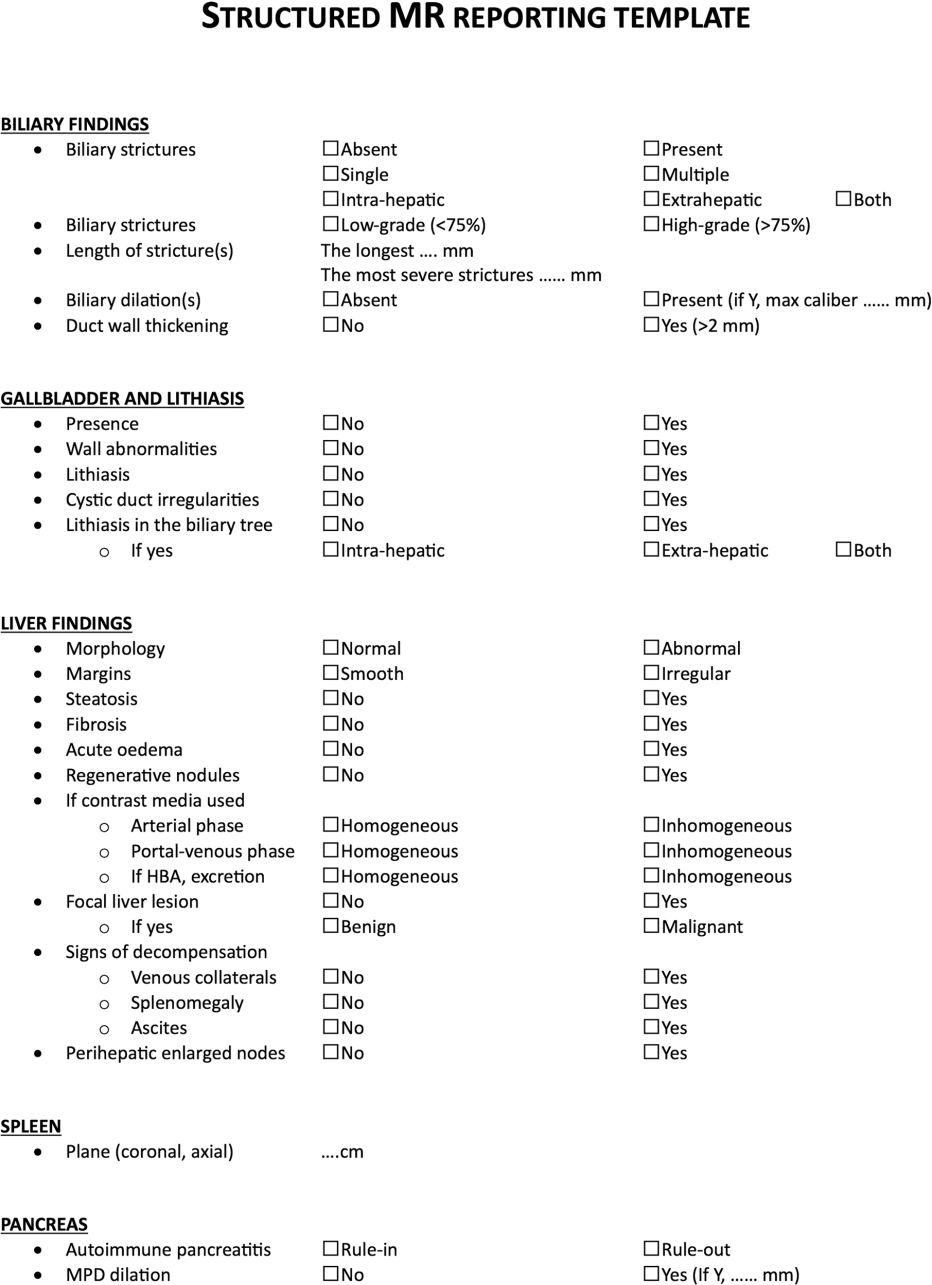
The structured MR reporting template

According to the final position statement, all panelists approved a structured reporting template for MR as depicted in Fig. 1.

## Discussion

This ESGAR position statement concerning patients with suspected or diagnosed large-duct PSC and their follow-up could be developed thanks to the prompt results in the voting process obtained from the work group expert panelists.

### General statements, patient preparation, and acquisition of MR protocol

MR, predominantly MRCP sequences, is highly useful in assessing PSC patients due to its non-invasive nature. In this setting, MR plays a fundamental role in monitoring disease progression, detecting complications, monitoring treatment response, and detecting cholangiocarcinoma.

Regarding the magnetic field, the panelists did not prefer 1.5 T or 3 T, underlying that the selection depends on local availability and personal choice [10]. Before starting the MR examination, patients should fast for at least 4 h and drink an oral T2-hypointense agent (e.g., pineapple or blueberry juice) [11].

MRCP is among the minimum required sequences for MR examination of patients with suspicion or known PSC. Regarding the MRCP sequence, the 3D-MRCP should always be preferred in comparison with 2D-MRCP, if feasible, because of the use of thinner slice thickness, which results in higher spatial resolution with excellent signal-to-noise ratio. Moreover, post-processing 3D reconstructions allows the evaluation of multiplanar reconstructions. Although 3D-MRCP can provide higher spatial resolution than 2D-MRCP, the sequence acquisition generally requires a significantly longer time, thus introducing the risk of motion artifacts [12]. To overcome these disadvantages, several approaches for reducing the acquisition time should be considered (e.g., 3D gradient and spin-echo, 3D balanced steady-state free precession (b-SSFP), or fast-recovery fast spin-echo (FPFSE)). Moreover, deep-learning-based sequences can also help overcome these issues [13].

The 2D-MRCP sequences can be performed as a stand-alone if the 3D-MRCP acquisition shows respiratory movement artifacts because the patient cannot breathe consistently or the respiratory triggering is not feasible. Orthogonal coronal plane acquisition covering most of the liver, anterior to posterior, should be preferred for adequate evaluation of peripheral ducts [14].

Moreover, acquiring T1WI IP/OP (or DIXON, if available) in the axial plane has been recommended to obtain information on hepatic and pancreatic parenchyma. T1WI may also aid in detecting intrahepatic biliary stones, which often show high signal intensity on T1WI [15].

Finally, DWI sequences acquired in the axial plane using both low and high b-values (at least 3) should be considered routinely, as they provide additional information regarding active inflammation and distribution of fibrosis. Furthermore, DWI increases sensitivity in detecting malignancy, such as cholangiocarcinoma and other focal liver lesions [16, 17].

Although there is no strong scientific evidence regarding the usefulness of intravenous contrast media injection, its administration may help improve the detection and characterization of focal benign or malignant lesions and peribiliary inflammatory changes. The panelists reported a fair agreement on this topic. If contrast media is administered, hepatobiliary contrast agents (HBAs) should be preferred over extracellular ones (ECAs), mainly due to their bimodal properties [18].

### Biliary findings

The identification of multifocal strictures is fundamental for the diagnosis of PSC. They may or may not cause upstream biliary dilation and may involve only a part or the entire length of the duct [19]. As the interpretation of the images may be subject to high inter-observer variability, biliary findings must be concisely reported.

The length of the most severe stricture(s) and dilation(s) need to be reported and measured, preferably on the coronal planes [20]. Usually, the typical intrahepatic bile duct walls are thin or unrecognizable on axial or coronal MR images. Therefore, any mural thickness > 2 mm should be considered abnormal, regardless of the site. Thickened bile duct walls can be measured on T2WI and T1WI after contrast media injection, if available.

The relationship between gallbladder and PSC has not been well-investigated or well-documented [21]. PSC-related inflammation and scarring can affect the entire biliary system, including the cystic duct, thus impacting bile flow and gallbladder distension [22].

### Findings of liver, spleen, and pancreas

The distortion of the liver morphology, characterized by a rounded or spherical shape caused by hypertrophy of the caudate lobe and atrophy of the right posterior segments, is still considered a typical finding of PSC progressing to cirrhosis [23].

Polygonal areas of confluent hepatic fibrosis demonstrate an intermediate signal on T2WI, matching the hypointense appearance on the portal-venous and HBP phases [17, 24]. In this setting, the panelists disagreed on MR’s superiority in identifying fibrosis compared to liver biopsy.

The panelists recommended detecting and characterizing regenerative nodules, particularly the macronodular ones (> 3 mm). They may develop in the entire liver parenchyma and appear hypointense on T1WI and T2WI, showing neither diffusion restriction nor abnormal contrast enhancement [25].

Portal vein caliber has been used in the past as a hallmark of chronic liver disease progressing to decompensation. However, other non-invasive signs are used to evaluate compensated advanced chronic liver disease. Endorsing them, the panelists did not recommend measuring portal vein diameter. On the other hand, the development of collateral vessels is a usual finding in the advanced stage of chronic liver disease [26]. Due to the clinical significance, most panelists underlined the importance of reporting them.

Splenomegaly, mainly due to portal hypertension, even if not clinically significant, may occur. Assessment of splenic length and/or volume may be used to predict adverse clinical outcomes and prognosis [27].

When evaluating the pancreas in patients with suspected chronic cholangiopathy, the panelists recommend ruling out imaging features of autoimmune pancreatitis. Similarly, it is paramount to rule out IgG4-related cholangiopathy and pancreatitis by combining radiological and clinical data [28]. Overall, in the case of a concomitant pancreatic involvement, decreased signal intensity of the pancreatic parenchyma on fat-sat T1WI can be detectable. In some cases, dilation of the main pancreatic duct has also been reported [29].

### Complications

Bacterial cholangitis may represent a complication in PSC patients and can occur spontaneously after bacterial overgrowth in obstructed bile ducts. The presence of biliary stones and biliary strictures enhances the risk of superimposed infection. The delayed or inappropriate treatment of bacterial cholangitis may lead to increased pressure in the biliary system, triggering increased permeability and necrosis of the biliary tree and extension of infection into the liver parenchyma [1].

The panelists recommended MR with MRCP as the first-line imaging technique to detect benign complications, such as cholangitis, in PSC patients. Endoscopic management is recommended in the case of clinically significant high-grade biliary stricture.

Hepatobiliary malignancies are a significant PSC-related complication. Cholangiocarcinoma is the most serious complication of long-standing PSC, occurring in up to 10-15% of patients [30]. Perihilar cholangiocarcinoma is the most prevalent hepatobiliary malignancy and the leading cause of mortality.

Recognizing cholangiocarcinoma can be challenging since both benign and malignant strictures may appear similarly on MR and MRCP. To detect it early, regular screening of PSC patients is proposed with CA 19.9 measurements every 6 months and MR with MRCP every year. The panelists recommend combining imaging data with serum markers.

Gallbladder cancer (GBC) and hepatocellular carcinoma (HCC) may also occur in the setting of PSC. The prevalence of GBC among those with PSC is estimated to be 2%. The risk of HCC in PSC has been reported to be relatively low. Longitudinal data from the US of 830 people with PSC detected 20 (2.4%) patients with HCC during a 9.5-year follow-up. HCC in patients with PSC should be carefully evaluated to exclude the possibility of cholangiocarcinoma. Once HCC is pathologically confirmed, patients should be offered therapeutic options according to standard guidelines [31, 32].

### Advanced imaging techniques

When using a hepatobiliary contrast agent, liver function can be assessed in the hepatobiliary phase (i.e., early changes of patchy inflammation possibly preceding the formation of fibrotic scarring and may serve as a marker for liver fibrosis). Bastati et al [33] have developed the functional liver imaging score (FLIS) based on three simple visual features: liver enhancement, biliary excretion, and portal vein signal intensity. These imaging criteria could also be applied to PSC to acquire functional information about the biliary tree. However, on this topic, the panelists recommended that further prospective and more extensive studies should be performed to validate these results before applying them to PSC patients in the clinical routine.

No agreement was reached concerning the application of T1 mapping in PSC patients [34]. Magnetic resonance elastography (MRE) was not considered helpful for PSC in everyday clinical practice. It is well known that MRE has several advantages over traditional imaging techniques for assessing tissue stiffness [35]. It is non-invasive, allows quantitative assessment, and can provide a whole-liver tissue stiffness evaluation rather than relying on localized palpation or biopsies. However, the lack of clinical studies in PSC patients does not currently allow any recommendation from the panelists [35, 36].

Although shear wave elastography showed promising results in PSC patients [37], the panelists did not collect sufficient studies to judge or include them in the present statement.

Finally, the panelists do not recommend radiomics and artificial intelligence software (e.g., MRCP+) [38], even if promising results have been reported in the recent international literature. Further prospective and more extensive studies should be performed before considering them robust and applicable in clinical settings.

### Prognostic scores

Different prognostic models have been developed to provide risk estimation for clinical outcomes in PSC [39]. In this setting, the panelists’ primary recommendation was that all scores, focusing on the ANALI, suffer from moderate to low inter-observer agreement.

The ANALI scores accurately predicted 4-year radiological progression from baseline with subsequent validation in a retrospective multicenter study [40]. Although Grigoriadis et al [41] found poor to moderate inter-reader agreement, they also confirmed a correlation with clinical outcomes, highlighting MR value in determining PSC prognosis.

Consequently, prognostic scores should be implemented in clinical practice with caution.

## Conclusions

This statement paper from the ESGAR working group provides recommendations on how and when we should perform MR in large-duct PSC patients. It also provides a structured reporting template that will improve quality care and communication among clinicians.

## Abbreviations

DWI: Diffusion-weighted imaging
ERCP: Endoscopic retrograde cholangiopancreatography
MRCP: Magnetic resonance with cholangiopancreatography sequences
PSC: Primary sclerosing cholangitis
T1WI: T1 weighted imaging
T2WI: T2 weighted imaging
